# Deficit of corpus callosum axons, reduced axon diameter and decreased area are markers of abnormal development of interhemispheric connections in autistic subjects

**DOI:** 10.1186/s40478-018-0645-7

**Published:** 2018-12-19

**Authors:** Jarek Wegiel, Wojciech Kaczmarski, Michael Flory, Veronica Martinez-Cerdeno, Thomas Wisniewski, Krzysztof Nowicki, Izabela Kuchna, Jerzy Wegiel

**Affiliations:** 1 Department of Developmental Neurobiology, New York State Institute for Basic Research in Developmental Disabilities, 1050 Forest Hill Road, Staten Island, NY 10314 USA; 2 Research Design and Analysis Services, New York State Institute for Basic Research in Developmental Disabilities, Staten Island, NY 10314 USA; 3 Pathology and Laboratory Medicine, Institute for Pediatric Regenerative Medicine, MIND Institute, University of California, Davis, CA USA; 4 Departments of Neurology, Pathology and Psychiatry, NYU Langone Medical Center, New York, NY USA

**Keywords:** Autism, Neuropathology, Corpus callosum, Axon, Electron microscopy, Morphometry

## Abstract

**Introduction:**

In autism spectrum disorder, lack of coherence and of complex information processing, and narrowly focused interests and repetitive behaviors are considered a sign of long-range underconnectivity and short-range overconnectivity. The goal of this morphometric study of five anatomically and functionally different segments of the corpus callosum (CC) was to establish patterns of differences between long-range interhemispheric connections in nine neurotypical and nine autistic subjects.

**Results:**

Electron microscopy revealed a significant reduction in average axon diameter and axon cross-sectional area in autistic subjects, and reduction in CC segment–specific diversification of connections of functionally different cortical regions. The study shows an increase in the percentage of small diameter axons (< 0.651 μm) and a decrease in the percentage of axons with large diameter (> 1.051 μm). The total number of small-diameter axons is reduced in segment I and III by 43% on average. The number of medium- and large-diameter axons is reduced in all five CC segments by an average of 49 and 72%, respectively.

**Conclusions:**

The detected pattern of pathology suggests a failure of mechanisms controlling guidance of axons during development leading to axonal deficit, and failure of mechanisms controlling axon structure. A reduction in axon diameter may affect the velocity and volume of signal transmission, and distort functional specialization of CC segments. Significant deficits in axon number and reduction in axon size in all five CC segments appear to be substantial components of brain connectome integrity distortion which may contribute to the autism phenotype.

## Introduction

### Developmental abnormalities of brain connectivity in autism

Autism spectrum disorders (ASDs) are debilitating neurodevelopmental conditions characterized by impairments in social interactions and communication combined with restricted interests and repetitive behaviors [3]. Despite the genetic and clinical heterogeneity of ASDs, Geschwind and Levitt [16] hypothesized that a common feature of the brain across the many forms of autism is developmental disconnection, resulting from the failure to develop normal connections between higher-order association areas of the temporal, parietal, and frontal cortices. Just et al. [28] formulated an underconnectivity theory, whereas Belmonte et al. [6] suggested a combination of reduced long-distance, and increased local connectivity in ASD. Functional [27, 30, 34] and structural [5, 31] neuroimaging findings support the concept of disconnection [12, 15] and underconnectivity [25, 28]. The first postmortem study of axonal connections in autism published by Zikopoulos and Barbas [61] revealed disconnection of long-distance pathways and excessive connections between neighboring areas in the white matter adjacent to the anterior cingulate cortex, orbitofrontal cortex and lateral prefrontal cortex suggesting contribution of these developmental defects to diagnostic behavioral alterations in autism. The combined effect of developmental abnormalities reported in imaging and postmortem studies is loss of integrity in brain connectivity, which appears to be essential for deficits of higher-order social, emotional, and communication activities in autistic subjects [46, 61].

### Corpus callosum (CC) developmental abnormalities in autism

The CC, the largest fiber tract in the human brain [23, 45, 55], is a primary target for testing the hypothesis of long-range underconnectivity. The CC connects brain hemispheres and facilitates integration of sensory and motor information with executive functions, social interactions, and language [38]. Structural MRI of the brain of autistic subjects shows a thinner CC and reduced CC midsagittal area, suggesting a deficit of long-range interhemispheric axonal connections [9, 21, 27, 36, 41, 42]. Brain fiber tractography based on diffusion tensor imaging divided the human CC into five segments, with axons projected through the brain midline by neurons of the prefrontal (S I), premotor and supplementary motor (S II), motor (S III), sensory (S IV), and parietal, occipital, and temporal cortices (S V) [24]. Morphometry of the CC of neurotypical subjects reveals a segment-specific ratio of axons of different diameter. In the brains of control subjects, the percentage of small-diameter axons is the largest in S1 and decreases in posterior segments, whereas the percentage of medium- and large-diameter axons increases in the middle and caudal portions of the CC [45]. Axon diameter, cross-sectional area, and myelin sheath thickness correlate with the velocity and volume of signal transmission [39, 47, 48]. Therefore, a CC segment–specific combination of these structural characteristics reflects the function of axons connecting specialized cortical regions.

To explain clinical MRI characterization of developmental anomalies of interhemispheric connections, we performed the first postmortem morphometric light microscopy study of the CC of autistic individuals [56]. The study revealed a significant deficit in interhemispheric axonal connections and two types of developmental abnormalities contributing to this deficit: a topographically selective partial CC agenesis and topographically non-selective hypoplasia. The detected regional agenesis and hypoplasia in CC segments are signs of focal interhemispheric disconnection and diffuse underconnectivity in autism.

### CC segmentation

There are no macroscopic anatomical landmarks that delineate distinct callosal areas in a midsaggital CC cross-section of the human corpus callosum. Several geometric partitioning schemes have been designed including the commonly used Witelson [60] CC division into five vertical segments. However, this segmentation was based predominantly on non-human primate studies. In this study, Hofer and Frahm [24] CC segmentation based on human brain diffusion tensor imaging (DTI) was applied. DTI fiber tracking identifies axonal bundles that cross the CC midline and separates transcallosal fiber tracts with respect to their specific cortical projections. Hofer and Frahm classification distinguishes segment (S) I with prefrontal cortex projections, SII with premotor and supplementary connections, SIII with motor connections, SIV with sensory cortex connections, and SV with parietal, temporal and occipital connections. Segmentation of the CC from the most rostral to the most caudal end of the CC defines the rostro-caudal extent of each segment with SI - 17%; SII - 33%, SIII -17%; SIV -8%; and SV – 25%.

### Aims

The aims of this electron microscopy study of axons in all five structurally and functionally different segments in the CC of autistic and control subjects were (a) to define the range of deficits in the number of small-, medium-, and large-diameter axons in CC segments connecting functionally diverse cortical regions; and (b) to define distortion in the segment-specific ratio of axons of different diameters as a marker of loss of specialization of interhemispheric connectivity in autism.

## Materials and methods

### Cohort

This study of the corpus callosum in autistic subjects is a component of a project designed by several research groups and supported by Autism Speaks and the Autism Tissue Program. The overall goal was to overcome problems associated with scarcity of material for postmortem studies resulting in studies of 3–4 cases by different groups applying different clinical criteria and different methods of examination of a few brain regions. Screening of 18 pairs of autistic and control subjects identified 12 age- and sex-matched pairs corresponding to inclusion criteria. One brain hemisphere of these subjects was processed and embedded in celloidin for detection of global patterns of anatomical, cellular and subcellular changes in autism.

This electron microscopic study of the CC was performed on 9 pairs of autistic and control subjects with well-preserved CC ultrastructure. The demographics, information about intellectual deficits, seizures, and cause of death are summarized in Table 1. In both the autistic and control group, the same number of males (6) and females (3) was examined. The age of subjects with autism was 5 to 60 years (25.4 on average) and of control subjects 4 to 52 years (26.3 on average). The diagnosis of autism was confirmed with the Autism Diagnostic Interview, Revised [33]. Intellectual deficit ranging from mild to severe was reported in six autistic subjects, and seizures in five cases. The average postmortem interval (PMI) was 21.2 and 15 h in the autistic and control groups, respectively. Differences in age, PMI, brain weight, and brain weight loss during dehydration were not significant.

**Table 1:** Preserved tissue and clinical and postmortem records

Group and case ID	Sex	Age (y)	Intellectual deficit	Seizures age of onset	Cause of death	PMI (h)	BW (g)	Weight loss (%)
A1	F	5	–	–	Traumatic injuries	13	1390	52
A2	M	8	–	–	Rhabdomysarcoma	22	1570	45
A3	F	11	Mild	4.5 m	Seizure-related drowning	13	1460	52
A4	M	13	Severe	2 y	Seizure-related	8	1470	39
A5	F	21	Moderate	5 y	Seizure related resp. failure	50	1108	43
A6	M	23	Severe	23 y	Seizure-related resp. failure	14	1610	60
A7	M	36	Severe	–	Cardiac arrest	24	1480	44
A8	M	52	–	–	Heart failure	11	1324	53
A9	M	60	Moderate	3 y	Pancreatic cancer	26	1210	38
Mean ± SD		25.4 ± 19.8				21.2 ± 13.6	1402 ± 164	47.3 ± 7.3
C1	F	4	–	–	Acute bronchopneumonia	17	1530	49
C2	M	14	–	–	Electrocution	20	1464	44
C3	F	15	–	–	Traumatic injuries	9	1250	49
C4	F	20	–	–	Traumatic injuries	9	1340	52
C5	M	23	–	–	Ruptured spleen	6	1520	41
C6	M	29	–	–	Traumatic injuries	13	1514	49
C7	M	32	–	–	Asphyxia	24	1364	42
C8	M	48	–	–	Heart atherosclerosis	24	1412	39
C9	M	52	–	–	Heart atherosclerosis	13	1430	48
Mean ± SD		26.3 ± 15.8				15 ± 6.6	1424 ± 94	45.9 ± 4.5

*A* autism, *C* control, *PMI* postmortem interval, *h* hours; Weight loss, decrease (%) of hemispheric brain sample weight during dehydration in ethyl alcohol; *SD* standard deviation

### Tissue sampling for estimation of CC area, number of axons, and EM-based axon morphometry

The brain hemisphere was fixed in 10% buffered formalin for at least 3 months, washed for 24 h in water to remove fixer, dehydrated in increasing concentrations of ETOH, embedded in celloidin [22] and cut into 200-μm-thick frontal hemispheric sections. For morphometric study, a 2-mm wide CC strip was cut off from every twelfth coronal hemispheric section, impregnated with 2% osmium tetroxide, dehydrated and embedded in Epon. Resin blocks were cut into 1-μm-thick sections. Samples were oriented to cut axons perpendicularly to axon long axis and stained with a 2% solution of p-phenylenediamine (PPD). Every 60th section was used to determine the entire midsagittal area of the CC and each of the five segments, whereas 21 equidistant sections were used for estimation of the total number and numerical density of axons under a light microscope. The results were summarized in our previous paper [56].

### Electron microscopy study

**Fig. 1:**
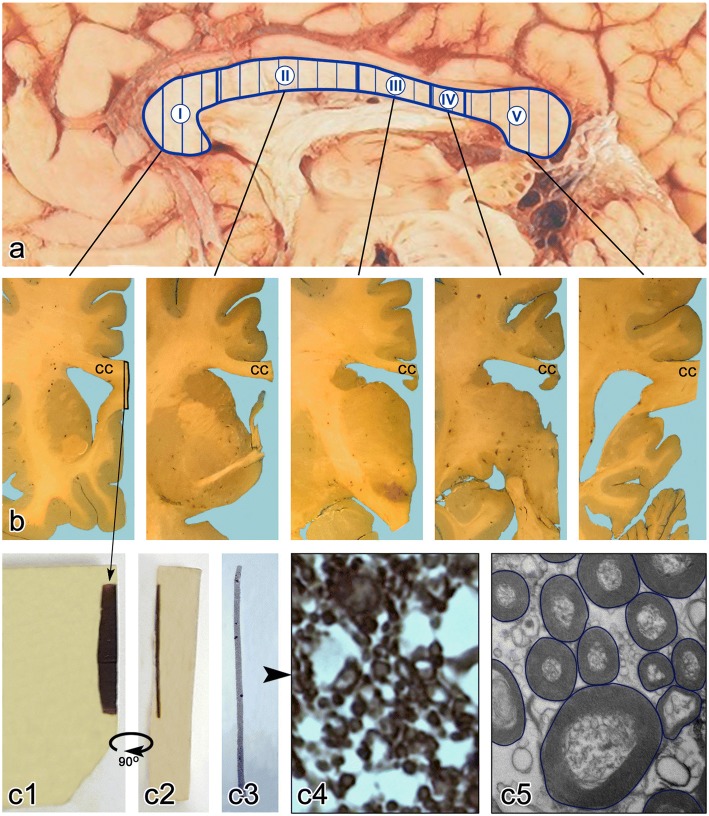
CC sampling for light and electron microscopy study. Diffusion tensor imaging and fiber tractography-based vertical partitioning of the CC into five segments corresponding to interhemispheric cortical connections (**a**). Celloidin embedded brain hemisphere was cut into 200-μm-thick gapless serial sections (**b**). From thirteen equidistant CC samples representing all segments (**b**) a 2-mm-wide CC sample was cut off and embedded in Epon (**c1**). Semithin sections were cut (**c2**) and stained with PPD (**c3**) for light microscopy morphometric studies [56]. For the EM morphometric study of axons Epon blocks were trimmed and 60-nm-thick sections were cut and stained with uranyl acetate. The calibrated electron micrographs obtained at 15,000x magnification were used for manual delineation of the axon border (**c4**) to estimate the diameter and crossectional area of the axon, as well as to estimate the thickness of the myelin sheath measured at four equidistant points. The measurements were performed with Image J software

Thirteen Epon blocks represented the entire rostro-caudal extent of the CC in each brain of nine control subjects and nine autistic subjects, including three Epon blocks for S I, S II, and S V and two blocks for the shorter segments, III and IV (Fig. 1). For electron microscopy, Epon blocks were cut into 60-nm-thick sections using a Reichert Ultracut S (Leica, Austria) ultramicrotome and Diatome 45° knife (Diatome, U.S., Fort Washington, PA, USA), and collected on formvar-coated copper grids. Sections were stained with uranyl acetate and photographed at a magnification of 15,000x using a Hitachi H7500 transmission electron microscope with a CCD camera and Advanced Microscopy Techniques’ (AMT) Image Capture Engine (Danvers, MA). For each case, 12 electron micrographs were used for measurements of axon diameter, cross-sectional area, and myelin thickness. Background correction was applied to reduce risk of distortions during image analysis. For morphometric analysis, the images were saved in the jpeg format.

### Axon measurements

The study of CC axons was limited to myelinated axons, which are more common (comprising approximately 90% of all axons [32] and are better preserved than non-myelinated axons [45]. On average in the CC of each control and autistic subject, 1676 and 1486 axons, respectively, were measured. The total number of axons measured in nine control subjects was 15,085, and in nine autistic subjects 13,376. Oversampling was necessary to detect significant differences in all CC segments, including the smallest sector IV representing only 8% of the linear rostro-caudal CC extent [24]. Axons were measured manually by delineation of the outer axon myelin contour (Fig. 1). Image J software provided measurements of axon diameter (Feret’s diameter, μm) and area (μm^2^). Average myelin thickness of axons was estimated by measuring the myelin sheath thickness at four equidistant points.

### Statistical analysis

Analyses of axonal diameter, cross-sectional area, and myelin thickness across diagnostic groups and segments of the CC were done in repeated-measures ANOVAs, using the conservative Greenhouse-Geisser adjustment for the non-sphericity of the dependent variables across segments [19]. Comparisons of percentages of small-, medium-, and large-diameter axons across five segments were done using the wsanova routine [17]. *Post-hoc* analyses used one-way ANOVAs and T-tests. All analyses were performed using Stata version 15.1 [51]. To address the potential increase in the likelihood of Type II errors (false positives) with multiple comparisons, we have used control of the False Discovery Rate (FDR) [7] rather than performing Bonferroni-type corrections of our criterion for significance [18].

## Results

### Differences between distribution of axon diameter in control and autistic subjects

Characterization of axon diameter distribution was based on comparison of an average of 335 axons per segment per control subject and 297 axons per segment per autistic subject. The reason for examination of fewer axons in the autistic group was lack of axons in S IV and a partial deficit in the caudal part of S III and the anterior part of S V in three autistic subjects with partial CC agenesis.

**Fig. 2:**
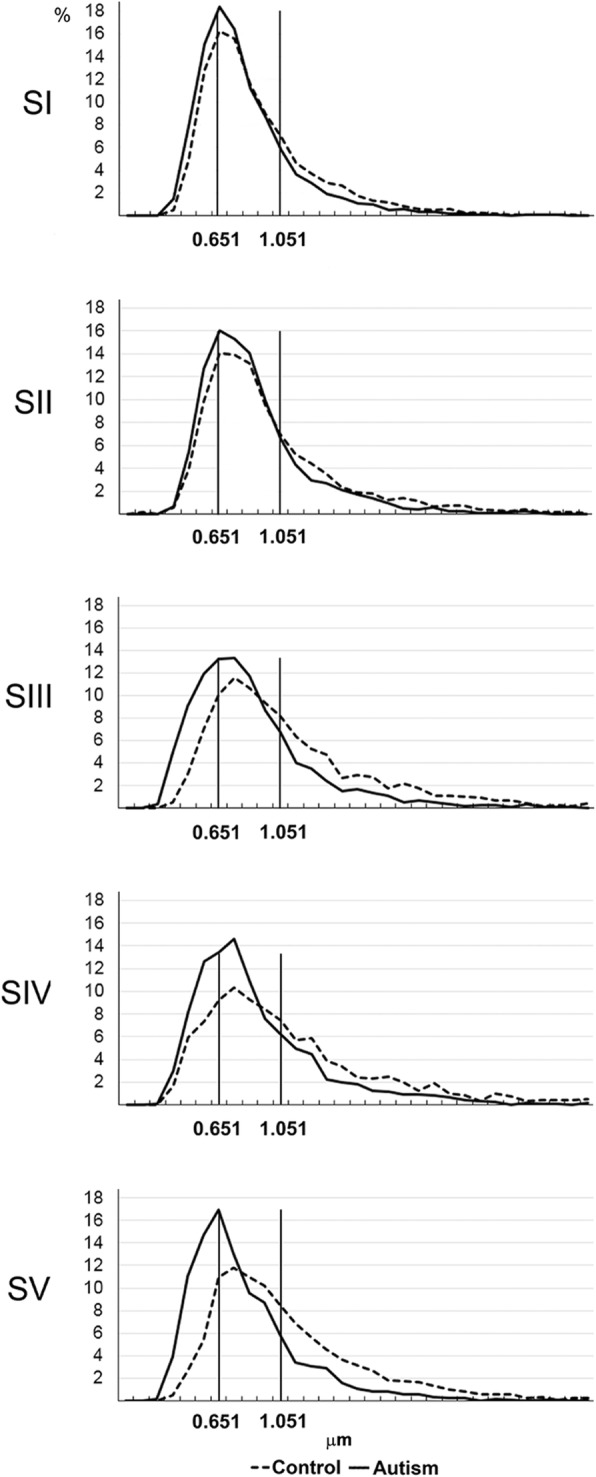
Differences between axon diameter distribution in autism and control. The distribution curves of axon diameter in all five CC segments in autistic and control subjects revealed a higher percentage of small-diameter axons and a lower percentage of large-diameter axons in autistic subjects, with more prominent differences in posterior S III– S V than in anterior S I and S II

The distribution curves for all five segments (Fig. 2) suggested that three intervals (< 0.651 μm; 0.651–1.051 μm and >  1.051 μm) identify differences in the prevalence of small-, medium-, and large-diameter axons. This classification was applied to compare the percentage of small-, medium-, and large-diameter axons in control and autistic groups. The axon diameter distribution curves for each of the five CC segments revealed an increased percentage of small-diameter axons and a decreased percentage of large-diameter axons in autistic subjects. The prevalence of small- and the deficit of large-diameter axons was relatively low in S I, but the difference became larger in the four posterior segments.

The detected differences among autistic subjects in the ratio of varying diameter axons in the CC segments reflected a distortion in structural and functional specialization of individual CC segments connecting functionally different cortical regions. To define interhemispheric connection abnormalities, inter-segmental differences in average axon diameter, cross-sectional area and myelin thickness, and the percentage and total number of small-, medium-, and large-diameter axons were characterized within the control and autistic groups.

### Differences in average axon diameter, cross-sectional area and myelin thickness in CC segments in neurotypical control and autistic subjects

An overall ANOVA analysis of axon measurements showed that in the autistic subjects, average axon diameter was significantly less than in the control group (F [3] = 18.31, *p* = 0.0001) and diameter differed across segments (F [6] = 7.32, Greenhouse-Geisser *p* = 0.0012) [19].

**Fig. 3:**
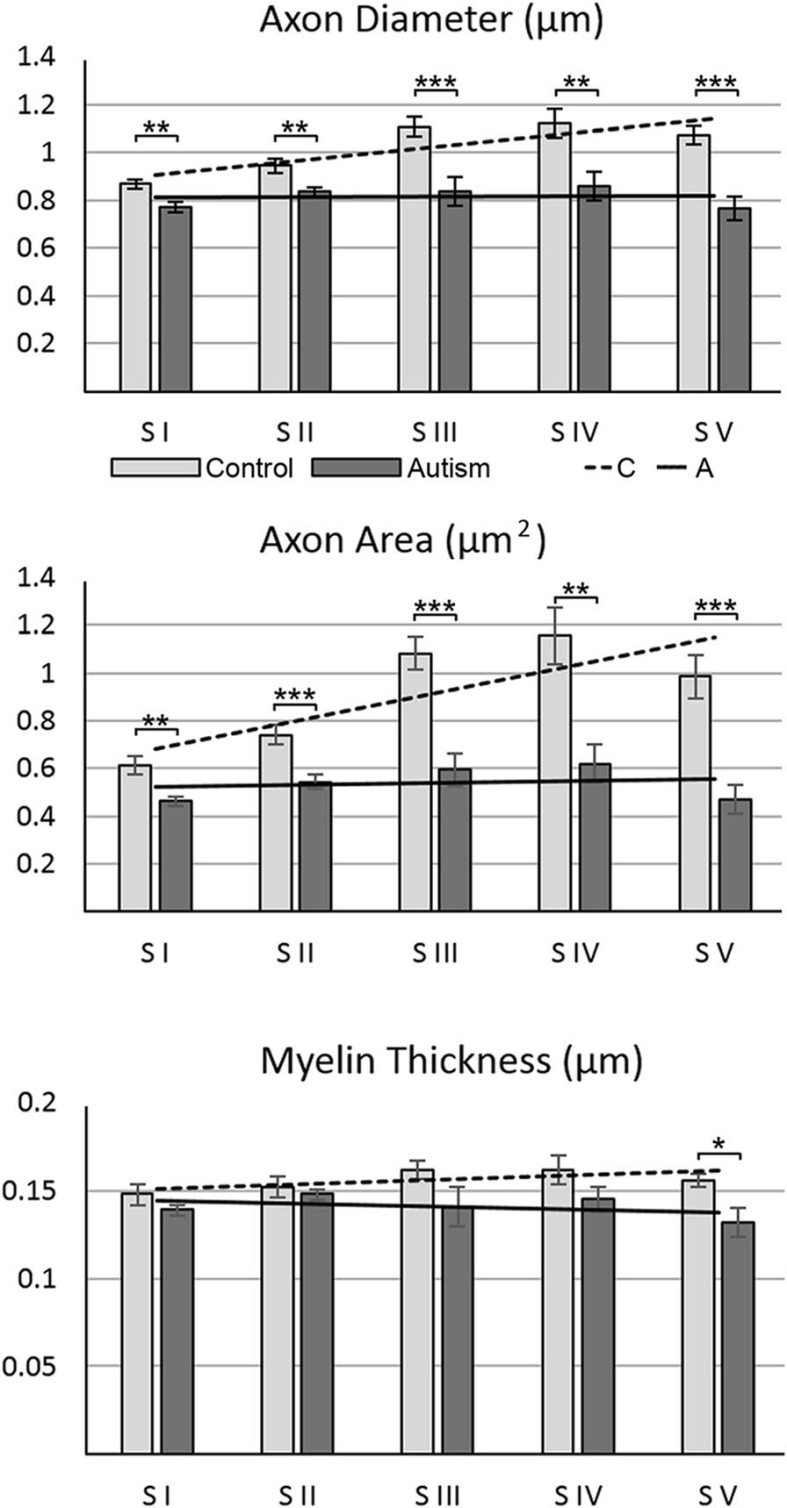
Axon diameter, cross-sectional area, and myelin thickness. In the CC of control subjects, axon diameter and cross-sectional area were the lowest in S 1 but increased significantly in S II–V, and the increment in segments varied in a broad range. In autistic subjects, the average axon diameter and cross-sectional area were significantly less than in the control group, and differences between segments became insignificant. In contrast to variations of axon diameter and cross-sectional area, the average thickness of myelin sheath was comparable in five segments of control subjects. In autistic subjects, significant reduction in myelin thickness was detectable only in S V

Within-segment ANOVA-based comparison of average axon diameter in autistic and control subjects revealed a significantly smaller diameter in autistic subjects, with the smallest difference in S I (11.2%) and a linear increase to a 28.6% deficit in S V (Table 2, Fig. 3). Reduced axon diameter is paralleled by a very significant deficit in axon cross-sectional area ranging from 24.6% in S 1 to 51.6% in S V in autistic subjects. Measurements of the thickness of the axon myelin sheath revealed insignificant deficits in S I–IV and a significant 15.8% (*p* <  0.018) deficit limited to SV.

**Table 2:** The difference in average axon diameter, midsagittal area, and myelin thickness in five CC segments in autistic (A) and control (C) subjects

Parameter	Group	CC Segment				
		S I	S II	S III	S IV	S V
Axon diameter (μm) (SE)	C	0.869 (0.025)	0.945 (0.026)	1.108 (0.036)	1.120 (0.058)	1.075 (0.043)
	A	0.772 (0.016)	0.836 (0.020)	0.837 (0.059)	0.858 (0.061)	0.768 (0.046)
	*p* <	0.005**	0.004**	0.001***	0.010**	0.0002***
	Difference	−11.2%	−11.5%	−24.4%	−23.4%	−28.6%
Axon area (μm^2^) (SE)	C	0.613 (0.042)	0.739 (0.039)	1.075 (0.068)	1.157 (0.117)	0.968 (0.086)
	A	0.462 (0.024)	0.543 (0.032)	0.594 (0.071)	0.620 (0.081)	0.469 (0.056)
	*p* <	0.006**	0.001***	0.0002***	0.005**	0.0002***
	Difference	−24.6%	−26.5%	−44.8%	−46.9%	−51.6%
Myelin thickness (μm) (SE)	C	0.149 (0.006)	0.153 (0.006)	0.162 (0.005)	0.163 (0.008)	0.157 (0.004)
	A	0.139 (0.003)	0.149 (0.003)	0.142 (0.011)	0.145 (0.007)	0.132 (0.008)
	*p* <	0.197	0.538	0.092	0.141	0.018*
	Difference	ns	ns	ns	ns	−15.8%

*CC* Corpus callosum, *SE* standard error, *ns* not significant

*p ≤ 0.05; **p ≤ 0.01; ***p ≤ 0.001

The second analysis, focused on detection of differences between axon diameters in individual segments of the CC in neurotypical control subjects, revealed significant intersegmental differences, with an increase of average axon diameter from 0.869 μm in S I to 1.108 μm in S III, 1.120 μm in S IV, and 1.075 μm in S V (Fig. 3, Table 2). Analysis showed significant increase of axon diameter in three posterior segments of control subjects (Table 3). The average axon area increased from 0.613 um^2^ in S I to 1.157 um^2^ in S IV and 0.968 μm^2^ in S V, and the increase was significant in these segments. The increase in axon myelin thickness was small and was significant only in S III. This pattern of significant inter-segmental differences reflects a specialization in CC connections of functionally different cortices in the human brain.

**Table 3:** Significance of difference of axon diameter, cross-sectional area, and myelin thickness between S I and other segments in the CC in autistic and control subjects (test for diversity of axons within segments)

Parameter	Group	S I/S II	S I/S III	S I/S IV	S I/S V
Axon diameter	C	0.055	0.0001***	0.003**	0.0004***
Axon cross-sectional area	C	0.049*	0.0004***	0.003**	0.0014***
Myelin sheath thickness	C	0.483	0.042*	0.101	0.200
Axon diameter	A	0.218	0.440	0.440	0.947
Axon cross-sectional area	A	0.174	0.174	0.174	0.931
Myelin sheath thickness	A	0.055	0.964	0.929	0.891

*P* values incorporate multiple-comparison adjustments to maintain a false discovery rate (FDR) of 0.05. *p ≤ 0.05; **p ≤ 0.01; ***p ≤ 0.001. *S* Segment, *CC* corpus callosum, *C* control, *A* autism

In contrast to the broad range of intersegmental differences in the control group, in autistic subjects axon diameter and area varied in a narrow range, and differences between segments were not significant (Table 3). The flat trend line reflecting a rather uniform diameter of axons and axon area in all segments in autistic subjects (Fig. 3) was a sign of lack of structural differentiation of axons in CC segments.

Autism was a less significant factor in myelin thickness than it was in diameter or area (F [3] = 5.42, *p* = 0.0233), while neither the segment nor its interaction with the diagnosis had a significant effect.

### Decrease in the percentage of small-diameter axons and increase in percentage of large-diameter axons in posterior segments of the CC in neurotypical control subjects

To learn which factors define the higher, segment-specific increase in average axon diameter in S II–V of control subjects, the percentage of small-, medium-, and large-diameter axons was determined in the five segments. The percentages of axons falling within each size range differed strongly by segment for controls (F [6] = 6.61, Greenhouse-Geisser *p* = 0.0005).

**Fig. 4:**
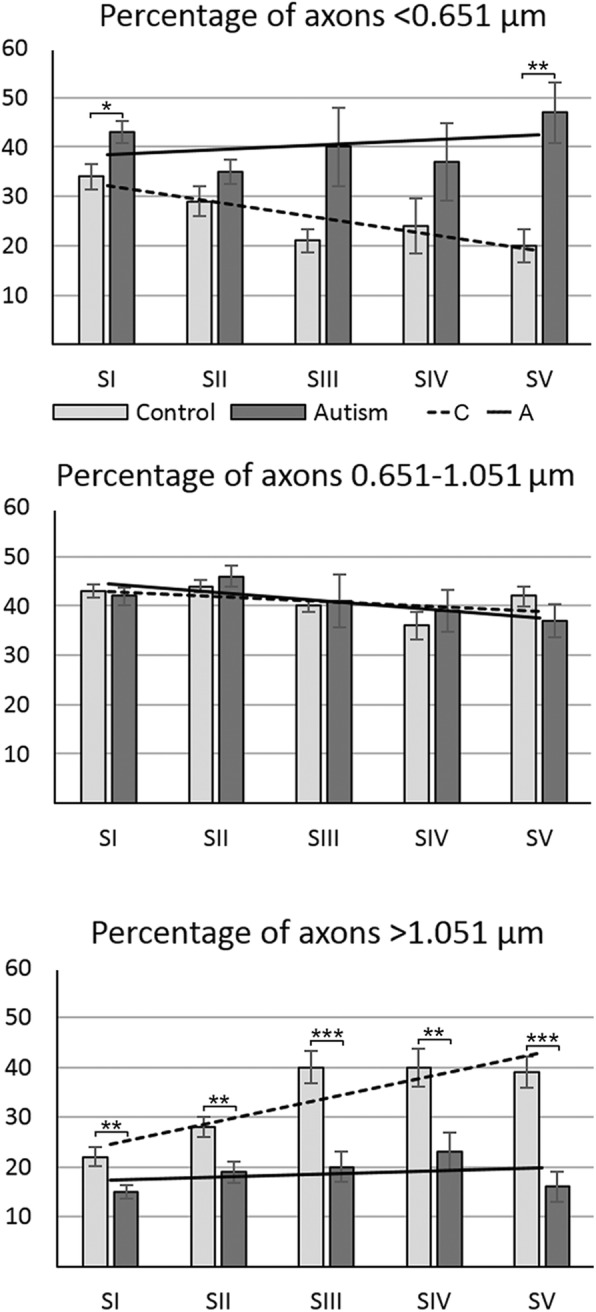
Changes of the percentage of small-, medium-, and large-diameter axons in autism. Estimation of the percentage of small- (< 0.651 μm), medium- (> 0.651–1.051 μm), and large-diameter (> 1.051 μm) axons revealed that three features characterized CC connectivity in neurotypical control subjects: significant decrease in the percentage of small-diameter axons in posterior segments, especially in S III–S V; increase in the percentage of large-diameter axons, especially prominent in S III–S V, and a broad heterogeneity of axon size in all segments. The sign of pathological remodeling of interhemispheric connections in CC segments in autistic subjects was stabilization of the percentage of small-, medium-, and large-diameter axons close to approximately 40, 40, and 20%, respectively. The effect of these changes in autistic subjects is the loss of structural diversity of axons typical for a normal brain, resulting in almost flat trend lines for the percentages of small-, medium-, and large-diameter axons in CC segments in autistic subjects

In control subjects, the percentage of small-diameter axons (< 0.651 μm) decreased from 34.6% in S I to 19.1% in S V (*p* <  0.002) (Tables 4, 5; Fig. 4). The opposite trajectory was detected for large-diameter (> 1.05 μm) axons, with an increase in the percentage of these axons from 22.2% in S I to 40.2% in S IV (*p* <  0.0015). In contrast to the distinct trajectories of small- and large-diameter axons, the percentage of medium-diameter axons varied in a narrow range from 35.4% (S IV) to 43.8% (S II). These differences were not significant, and there was no detectable trend in the rostro-caudal extent of the CC (Table 5).

**Table 4:** Different patterns of distribution of the percentage (± SE) of small-, medium-, and large-diameter axons in segments I–V in autistic and control subjects

Axon diameter (μm)	Group	Percentage of axons in CC segments				
		S I	S II	S III	S IV	S V
< 0.651	C	34.6 (2.6)	28.0 (3.0)	20.5 (2.4)	24.3 (5.6)	19.1 (3.4)
	A	43.1 (2.2)	35.0 (2.4)	38.9 (7.9)	37.8 (7.8)	46.6 (6.2)
	*p* <	0.027*	0.093	0.058	0.175	0.0014**
	Diff.	+ 24.42%	ns	ns	ns	+ 143.7%
0.651–1.051	C	43.1 (1.3)	43.8 (1.3)	39.8 (1.2)	35.4 (2.8)	41.6 (2.0)
	A	42.2 (1.8)	45.8 (2.1)	40.8 (5.3)	39.0 (4.2)	36.9 (3.4)
	*p* <	0.696	0.429	0.858	0.472	0.261
	Diff.	ns	ns	ns	ns	ns
> 1.051	C	22.2 (1.9)	28.2 (2.1)	39.6 (3.3)	40.2 (3.8)	39.2 (3.2)
	A	14.7 (1.3)	19.0 (2.1)	20.3 (3.0)	23.1 (3.8)	16.4 (3.1)
	*p* <	0.005**	0.008**	0.007***	0.010**	0.0001***
	Diff.	−34.02%	−32.52%	−48.82%	−42.52	−58.2%

*SE* Standard error, *CC* corpus callosum, *S* segment, *C* control, *A* autism; Diff., difference; ns, not significant. Standard errors in parentheses

**Table 5:** Significance of difference in the percentage of small-, medium-, and large-diameter axons in segment I compared to four other segments in the CC in autistic and control subjects

Axon diameter (μm)	Group	S I/S II	S I/S III	S I/S IV	S I/S V
< 0.651	C	0.073	0.002**	0.073	0.002**
0.651–1.051	C	0.646	0.224	0.052	0.616
> 1.051	C	0.048*	0.0002***	0.0015**	0.0002***
< 0.651	A	0.028*	0.663	0.663	0.663
0.651–1.051	A	0.232	0.745	0.404	0.232
> 1.051	A	0.221	0.221	0.221	0.684

*CC* corpus callosum, *S* segment, *C* control, *A* autism. *P* values incorporate multiple-comparison adjustments to maintain a false discovery rate (FDR) of 0.05. *p ≤ 0.05; **p ≤ 0.01; ***p ≤ 0.001

### Reverse trajectory in the CC of autistic subjects with an increase in the percentage of small-diameter axons and decrease of large-diameter axons in the posterior CC segments (Fig. 4, Tables 4, 5)

A comparison of the percentage of small-diameter axons in control and autistic subjects revealed an increased contribution of small-diameter axons in all segments of autistic subjects, with a significant increase limited to S I (+ 24%; (*p* <  0.027) and S V (+ 143%; *p* <  0.0014). Comparison of large-diameter axons revealed a reverse trend, with a deficit of large axons in all CC segments of autistic subjects, ranging from − 34.02% in S I (*p* <  0.005) and − 58.2% in S V (*p* <  0.0001).

Table 5 summarizes the results of analyses focused on intersegmental differences in the ratio of axons with different diameters. In neurotypical subjects, a decrease in percentage of small-diameter axons was observed in all segments, but a significant decrease was detected in S III and S V. The increase in percentage of large-diameter axons was significant in all posterior segments in the control group. In the autistic group, the percentages of axons falling within each size range do not show a significant difference (F [6] = 0.99, *p* = 0.4310). Table 5 illustrates the absence of intersegmental differences in all CC segments in autistic subjects.

This pattern was the product of a significant developmental pathology of the CC of autistic subjects documented with an almost flat trendline in the percentage of small-, medium-, and large-diameter axons (Fig. 4). These flat trendlines distinguished the CC of autistic subjects from a very consistent and significant decrease in the percentage of small-diameter axons and consistent and significant increase in the percentage of large-diameter axons in the posterior segments of the CC of neurotypical subjects. The results reflected an almost total loss of structural and functional differentiation of individual segment connections critical for normal human brain function.

### The dominant role of axon number developmental deficits in a significant distortion of connectivity in all five CC segments in autistic subjects

The major factor defining structure and function of interhemispheric connectivity was the developmental focal and diffuse deficit of axons in all segments and in the entire CC of autistic subjects. The total number of myelinated axons was 49.42% less in the autistic (44.304 million) than in the control group (89.655 million) (Table 6).

**Table 6:** The difference between number (million ± SE) of axons in five CC segments in autistic and control subjects

Group	S I	S II	S III	S IV	S V	Total number
C	25.900 (1.891)	24.944 (1.175)	9.878 (1.417)	3.711 (0.326)	25.222 (2.518)	89.655 (4.186)
A	10.933 (2.010)	15.500 (1.642)	3.211 (0.644)	1.360 (0.441)	13.300 (1.618)	44.304 (4.885)
*p* <	0.0001***	0.0003***	0.0012**	0.0006***	0.0011**	0.0001***
Differ.	−57.79%	−37.87%	67.49%	−63.35%	−47.27%	−49.42%

*SE* standard error, *CC* corpus callosum, *S* segment, *C* control, *A* autism; Differ., difference. Standard errors in parentheses. **p ≤ 0.01; ***p ≤ 0.001

**Fig. 5:**
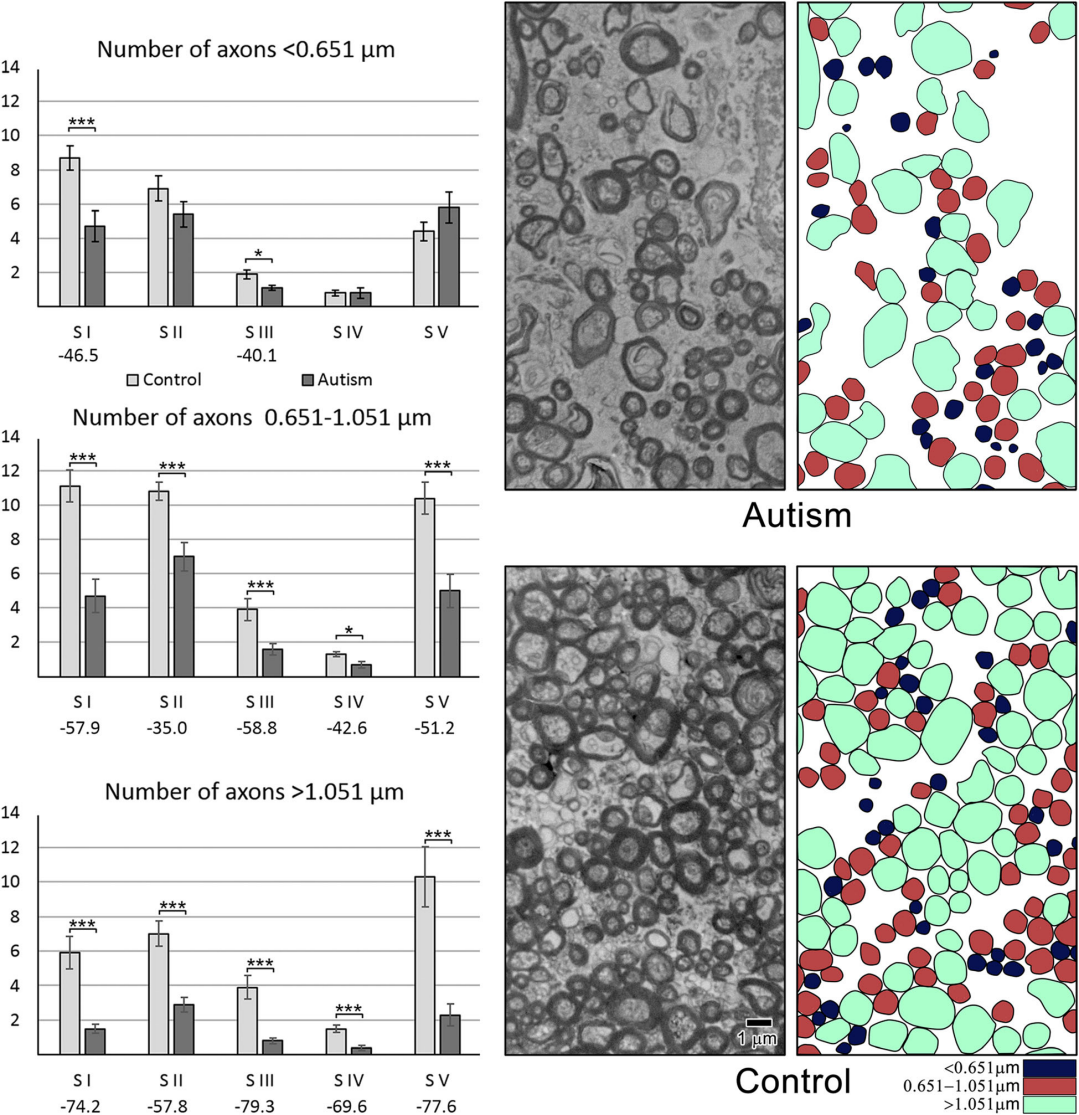
Segment-specific deficits in the number of small-, medium-, and large-diameter axons in autism. Estimation of total number (millions) of axons in the CC of control and autistic subjects revealed significant deficit in total number of small-diameter axons in S I (46.5%) and S III (40.1%) in autistic subjects. The dominant feature of all five segments was 49% deficit of medium-diameter axons and 72.6% deficit of large-diameter axons in autistic subjects. Electron micrographs and respective plots of axons illustrate deficit of small-, medium-, and large-diameter axons in CC genu (S I) in autistic subject compared to control subject

The estimates of the total number of small-diameter (< 0.651 μm) axons revealed a significant reduction in S I and S III by 46.5 and 40.1%, respectively (Table 7). Evaluation of the total number of large-diameter (> 1.05 μm) axons revealed a very consistent and significant deficit in all five segments in autistic subjects, by 72.6% on average. The number of medium-size axons (0.651 to 1.051 μm in diameter) was also reduced significantly in all five segments, by 49.0% on average (Fig. 5).

**Table 7:** The difference between number (± SE) of small-, medium-, and large-diameter axons in five CC segments in autistic and control subjects

Axon diameter	Group	S I	S II	S III	S IV	S V
< 0.651	C	8.789 (0.711)	6.955 (0.745)	1.924 (0.259)	0.862 (0.187)	4.427 (0.568)
	A	4.701 (0.890)	5.422 (0.742)	1.153 (0.150)	0.817 (0.291)	5.881 (0.885)
	*p* <	0.0025	0.164 (ns)	0.0253	0.8916 (ns)	0.1860 (ns)
	Diff.	−46.5%		−40.1%		
> 0.651 < 1.051	C	11.177 (0.930)	10.889 (0.530)	3.967 (0.639)	1.319 (0.144)	10.405 (0.934)
	A	4.704 (0.972)	7.083 (0.836)	1.633 (0.332)	0.757 (0.178)	5.084 (0.974)
	*p* <	0.0002	0.0014	0.0071	0.0294	0.0012
	Diff.	−57.9%	−35.0%	−58.8%	−42.6%	−51.2%
> 1.051	C	5.933 (0.951)	7.099 (0.735)	3.985 (0.679)	1.529 (0.231)	10.389 (1.749)
	A	1.527 (0.275)	2.993 (0.438)	0.825 (0.183)	0.465 (0.159)	2.334 (0.644)
	*p* <	0.0015	0.0002	0.0014	0.0049	0.0015
	Diff.	−74.2%	−57.8%	−79.3%	−69.6%	−77.6%

*SE* standard error, *CC* corpus callosum, *S* segment, *C* control, *A* autism; Differ., difference. Standard errors in parentheses

The study revealed multilevel consequences of two pathological developmental processes: those reducing the number of axons, and those reducing the diameter of axons in autistic subjects. The final product is a segment-specific deficit of small-, medium-, and large- diameter axons, and loss of the integrity of interhemispheric connections in autistic subjects.

## Discussion

Processing deficits in high-functioning autistic individuals are detected as shortages in integration of information and coordination of multiple neuronal networks [35, 36]. The CC, the largest human brain commissure, provides long-range interhemispheric connections integrating and synchronizing anatomically and functionally specialized cortices. The pattern of detected pathology of CC connections in autistic subjects indicates that these abnormalities are a product of failure of two types of mechanisms: (a) those controlling the number of interhemispheric connections, and (b) those controlling axon structure, including axon diameter and cross-sectional area. Failure of the first type of mechanism results in loss of, or reduction in the number of axons and underconnectivity affecting all major anatomical and functional cortical regions. Failure of the second type of mechanisms results in reduction of axon diameter and cross-sectional area in all five CC segments already affected by an axon deficit. Functional studies have revealed that decrease of axon diameter correlates with reduction in the velocity and volume of signal transmission [8, 40, 47, 48]. Our study of autistic subjects provides evidence for the: (a) reduction in the number of long-range axonal connections in the CC, (b) abnormal structure and function of preserved axons, and (c) partial loss of structural and functional specialization of CC segments and integrity of interhemispheric connections.

### Interhemispheric underconnectivity

Numerous structural MRI studies have revealed in autistic subjects a reduced size of the CC, and abnormal shape, including thinning of the CC, considered to be markers of abnormal interhemispheric connectivity [10, 14, 20, 21, 27, 29, 41, 50, 54]. The first postmortem study of 11 brains of autistic subjects, including nine brains examined in this ultrastructural study, expanded on MRI observations with light microscopy resolution and counts of axons in five CC segments [56]. In the examined brains of autistic subjects, two types of CC pathology contributed to interhemispheric underconnectivity: focal agenesis and diffuse hypoplasia. In three autistic subjects, total agenesis of S IV disconnected the sensory cortex, whereas partial agenesis of S III and S V partially disconnected the motor and parietal cortex. Partial agenesis of S I reduced interhemispheric connections of the prefrontal cortex. This topography revealed several CC segments with a high susceptibility to misguidance of axons and regional interhemispheric disconnection in autistic subjects. The second major cause of the deficit of interhemispheric connections detected in all examined autistic subjects was hypoplasia, defined by CC thinning, reduction of CC midsagittal area, and diffuse 62% reduction in the number of axons affecting all CC segments. The effect is loss of structural and functional integrity of interhemispheric connections, which may result in dysregulation of the velocity and volume of information transferred between hemispheres, loss of coherence and deficits in complex information processing in autistic subjects.

### Mechanisms contributing to underconnectivity and abnormal structure/function of axons

Two independent and methodologically different studies of genes involved in neurodevelopmental disorders identified robust, statistically significant evidence for convergence of the input of ASD risk genes in glutamatergic projection neurons in cerebral cortex layers 2/3 [37] and 5/6 [59]. Neuropathological studies of cortical neurons revealed that the most common neuron developmental anomalies reported in the cerebral cortex of autistic subjects are multiregional reduction of neuron soma size [26, 52, 53], minicolumn pathology always associated with a decrease in neuron size [11], and cortical dysplasia with smaller neurons [10, 57]. The link between reduced size of neurons and reduced axon diameter is strengthened by the fact that these reports were based on examination of brains described in the current CC study. Energy capacity estimated by percentage of axon volume occupied with mitochondria increases with axon diameter [39]. One may assume that the energy capacity of smaller-diameter axons is proportional to the energy capacity of a reduced volume of neurons. CC connection pathology might be an effect of developmental anomalies of both neurons and glia. Long-range interhemispheric connectivity is controlled in utero by brain midline glial cells at six decision points. Misguidance of axons at any of these points by factors independent of the axon may change the trajectory and target of the axon [4, 43, 44] and result in partial CC agenesis and hypoplasia in autistic subjects.

### Broad range of differences in axon diameter in CC segments in neurotypical subjects

Precision in synchronization of information transfer between hemispheres became more challenging with an evolutionary increase of brain size in apes and humans, which required both an increase in the velocity of long-distance interhemispheric conductivity and development of a variety of conduction velocities necessary for the functional diversity of human cortices and the brain [40].

Our study of neurotypical controls revealed a large range of axon diameters, reflecting a variety of conduction velocities, in each of the five CC segments, essential for cortices with different functions. Average axon diameter and cross-sectional area were the smallest in the first segment and increased in the posterior segments. The average diameter and cross-sectional area of axons in interhemispheric connections of the sensory cortex (S IV) were increased by 29 and 88%, respectively, in comparison to axons of the prefrontal cortex (S I).

Another feature of the CC in neurotypical controls was an increase in the percentage of large-diameter (> 1.051 μm) axons, known as high-velocity axons, from 22% in connections of the prefrontal cortex (S I) to 40–39% in connections of the motor (S III), sensory (S IV), and parietal, occipital, and temporal cortices (S V). High-transmission velocity across distant regions is essential for synchronization of the activity of remote neuronal circuits [49]. The percentage of small-diameter axons (< 0.651 μm) decreased from 34 to 29% in S I and II to approximately 20–24% in S III–S V. The axon composition detected in the examined control group resembled a higher percentage of small-diameter axons in the anterior CC and a higher percentage of large-diameter axons in the posterior portion of the CC reported in other studies of neurotypical subjects. This pattern reflected the structural and functional specialization of each segment of interhemispheric connections being critical for normal human brain function [1, 2, 45].

### Loss of diversity of axonal connections in five CC segments in autistic subjects

Velocity of signal transmission and firing rate correlate with axon diameter. Distribution of axon diameter, narrow or broad, symmetric or skewed, reflects the heterogeneity of information rates conveyed by the axon. A wide velocity range provides the wide range of timing necessary for synchronization of information delivery, processing, and response in an unaffected brain [39]. EM-based measurements of axons in autistic subjects revealed a reduction of average axon diameter by 12–29% and of average axon cross-sectional area by 25–52% in the rostro-caudal CC extent. These structural abnormalities resulted in a loss of the axon structural and functional diversification critical for regional and global cortical functionality.

Posterior CC segments are involved in the transfer of motor [55], somatosensory [13], and auditory and visual information [58] with a high demand for fast conduction and processing. However, reduction in the total number of axons with a diameter of 0.651–1.051 μm by an average of 49%, and of axons with a diameter >  1.051 μm by an average of 72.6%, is an indicator of a significant reduction in conductivity by both medium- and large-diameter axons. Moreover, the deficit of medium- and large-diameter axons affects not only posterior CC segments but also S I and S II connecting the prefrontal, premotor, and supplementary motor cortices. These deficits and the decreased diversification of interhemispheric connections may make a major contribution to the autism phenotype and comorbidities including intellectual deficits.

## Conclusions

This study revealed the complex nature of developmental defects affecting the structure and function of interhemispheric connections in autistic subjects. Agenesis and hypoplasia caused a severe reduction in the number of CC axons and loss of integrity of interhemispheric connectivity. Developmental anomalies of axons included a significant increase in the percentage of small-diameter and low-velocity axons, and a decrease in the percentage of large-diameter high-velocity axons in the CC of autistic subjects. The loss of structural and functional diversity in CC segments in autistic subjects reflected a loss of specialization of interhemispheric connections essential for higher-order functions and for integration of multisensory perception. In general, light and electron microscopy studies indicate that autism is associated with extensive developmental distortion of structure and function of interhemispheric connectivity.
